# Prevalence of Acute Tonsillitis and Its Association With Oral Hygiene Among the Population of Taif City, Saudi Arabia

**DOI:** 10.7759/cureus.55801

**Published:** 2024-03-08

**Authors:** Faisal A Alghamdi, Basil A Jawmin, Mohammed A Alghamdi, Meshari A Almalki, Yousef H Sabbagh, Ahmed A Aljemyie, Muath S Alotaibi, Rayan A Alolayani, Muhannad A Jawmin, Abdulaziz A Alghamdi, Turki A Althobaiti, Ahmed M Alqurashi, Khalid Hakami, Marwan F Alnofaie, Ayman A Atalla

**Affiliations:** 1 Faculty of Medicine, Taif University, Taif, SAU; 2 Department of Emergency Medicine, King Faisal Medical Complex, Taif, SAU; 3 Department of Inpatient Pharmacy, King Abdulaziz Medical City Jeddah, Makkah, SAU; 4 Department of Otolaryngology - Head and Neck Surgery, Rhinology Unit, Alhada Armed Forces Hospital, Taif, SAU; 5 Department of Otolaryngology - Head and Neck Surgery, King Faisal Medical Complex, Taif, SAU; 6 Department of Otorhinolaryngology, King Faisal Medical Complex, Taif, SAU; 7 Department of Family Medicine, College of Medicine, Taif University, Taif, SAU

**Keywords:** saudi arabia, middle-east, prevalence, sore throat, oral hygiene, acute tonsillitis

## Abstract

Introduction

Dental surfaces have dense bacterial deposits, and poor oral hygiene can exacerbate bacterial infections, causing acute tonsillitis. The study aims to quantify acute tonsillitis prevalence and assess its association with oral hygiene practices.

Methods

A descriptive cross-sectional study aimed to assess the prevalence of acute tonsillitis and its association with oral hygiene was conducted among adults aged 20 and above in Taif City, Saudi Arabia. Illiterates and those unwilling to participate were excluded. We employed an Arabic online self-administered questionnaire that was disseminated conveniently via Google Forms to social media assessing oral hygiene such as last dental visit, age at starting dental care, number of toothbrushes per day, frequency of toothbrush change, and duration of brushing teeth, and acute tonsillitis characteristics of the participants.

Results

About 393 participated in the study. Of them, 54% were aged 20-30, 53% were males, and 70% had a university education. The prevalence of acute tonsillitis was 64%. Approximately 28% reported dental clinic visits within three months, and 21% initiated oral hygiene practices at age 20. Among participants, 43% brushed twice daily, with 33% spending one minute and 43% two minutes. About 31% replaced toothbrushes every three months, while 23% acknowledged having bad breath. Experiencing bad breath, changing toothbrushes every three months, and having dental visits within less than three months were associated with having acute tonsillitis (p<0.05). However, regression analysis revealed that experiencing bad breath (OR: 2.11, 95% CI: 1.23, 3.70) was associated with a higher risk of acute tonsillitis, while less frequent toothbrush changes correlated with a lower risk (OR: 0.54, 95% CI: 0.30, 0.94).

Conclusion

This study revealed a substantial prevalence of acute tonsillitis among adults in Taif City. Oral care practices need improvement. There are significant associations between oral hygiene practices, bad breath, and the occurrence of acute tonsillitis. Addressing oral hygiene practices could be a key focus for preventative measures.

## Introduction

Tonsils at the upper aerodigestive tract serve as mucosa-associated lymphoid tissues, playing a crucial role in defending against numerous airborne and dietary pathogens by primarily engaging in humoral immune responses [1-3]. Tonsillitis, an inflammation of the tonsils, is mainly attributed to viral or bacterial infections, with viruses being the predominant cause [4]. 

Although there is not enough data on the global incidence of tonsillitis [5], in primary care, sore throat makes up 1.3% of outpatient visits [6]. 

Chronic tonsillitis (CT) is a long-term infection that occurs as a result of several repeated episodes of acute tonsillitis or as a result of a persistent infection that leads to chronic inflammation that is long-lasting and slowly progressing [7]. Acute tonsillitis is characterized by visible streaks of pus or cheesy material on the tonsillar surface, and the entire tonsil may become enlarged and hyperemic, suggesting an inflammatory process [8]. 

Symptoms of acute tonsillitis include fever, tonsillar exudates, sore throat, and tender anterior cervical chain lymphadenopathy [9]. 

Dental surfaces, characterized by dense bacterial deposits and non-shedding plaque or calculus, signify poor oral hygiene linked to caries, gingivitis, and periodontitis [10]. Poor oral hygiene is associated with internal and external disorders in the oral and oropharyngeal cavities [11]. Tonsillar actinomycosis, reported by Priyadharshini et al., suggests a potential link between dental plaque-related infections and tonsil issues [12-14]. 

Oral hygiene significantly prevents infections. Regular practices, including brushing and flossing, reduce bacterial load in the oral cavity. This directly relates to acute tonsillitis, where poor oral hygiene can exacerbate bacterial infections, impacting the frequency and severity of cases. Our study aims to quantify acute tonsillitis prevalence and assess its association with oral hygiene practices among adults in Taif City, Saudi Arabia. Through this exploration, we aim to provide insights into healthcare strategies tailored to this demographic, highlighting the critical role of oral hygiene in preventing acute tonsillitis.

## Materials and methods

Study design, population, and setting 

A descriptive cross‑sectional study aimed to determine the prevalence of acute tonsillitis and its correlation with mouth hygiene was conducted in Taif City. It is a city in the Makkah Region of Saudi Arabia. It is located at an elevation of 1,879 m on the slopes of the Hijaz Mountains. Taif City has a hot desert climate with hot summers and mild winters. 

The study included adults aged 20 years and above who lived in Taif City during the data collection period, regardless of gender and nationality, excluding illiterates and those who didn’t have smartphones due to the online nature of the questionnaire and who refused to be involved in the study. The sample size was calculated using the Raosoft sample size calculator. Considering a margin of error of 5%, an expected proportion of 50%, a 95% confidence interval, and a total population of 913,374 according to the 2022 Census [15], the minimum required sample size was 384. 

Data collection 

The data was collected using an online self-administered questionnaire designed according to study objectives and relative literature and validated by a pilot study after a thorough revision of a dentist and family medicine specialists. The questionnaire was disseminated conveniently via Google Forms to social media platforms, including only Taif City residents. 

The questionnaire contains three sections: the first includes demographic characteristics such as gender, age, marital status, nationality, and educational level. The second one assesses the oral hygiene of the participants, including last dental visit, age at starting dental care, number of toothbrushes per day, the use of mouthwash, frequency of dental floss use, frequency of toothbrush change, and duration of brushing teeth. The third section identifies acute tonsillitis characteristics such as history of acute tonsillitis, frequency of acute tonsillitis at age 20 years or above, antibiotic prescriptions, throat culture, fever with acute tonsillitis, swallowing difficulty, and the effect of acute tonsillitis on daily activity. The data was collected between June and August 2023. 

Data analysis 

Data was analyzed using the R statistics (R Foundation for Statistical Computing, Vienna, Austria). Means and standard deviations were calculated for numerical data, while frequencies and percentages were used to present categorical data. Fisher's exact and Pearson's chi-squared tests assessed the association between acute tonsillitis and oral hygiene. In contrast, multiple logistic regression with a backward selection method was used to identify if oral hygiene was a determinant of acute tonsillitis. A p-value less than 0.05 was set as the significance level of the study. 

Ethical consideration

Ethical approval was obtained from Taif University, which is accredited by the National Committee for Bioethics (HAO-02-T-105) with application no. 44-298.

## Results

Three hundred and ninety-three participated in the study, with 212 (54%) aged 20-30 years, 209 (53%) being males, 198 (50%) being singles, and 277 (70%) having university education (Table 1).

**Table 1:** Demographic characteristics of the participants ^1^N (%): number (percentage), and the total number of participants is 393.

Characteristic	N (%)^1^
Age	
20-30 years	212 (54%)
31-40 years	65 (17%)
41-50 years	92 (23%)
Above 50 years	24 (6.1%)
Sex	
Female	184 (47%)
Male	209 (53%)
Nationality	
Non-Saudi	38 (9.7%)
Saudi	355 (90%)
Marital status	
Single	198 (50%)
Married	176 (45%)
Divorced	8 (2.0%)
Widowed	11 (2.8%)
Educational level	
High school or lower	88 (22%)
Postgraduate	28 (7.1%)
University	277 (70%)

A significant majority, about 372 participants (95%), believed that maintaining oral hygiene plays a role in preventing diseases. However, 109 (28%) reported the last dental clinic visits within less than three months, 92 (23%) within 3-6 months, and 114 (29%) during the past year. 

In terms of the initiation of oral hygiene practices, 105 (27%) began in childhood, 141 (36%) in adolescence, and 82 (21%) at the age of 20, and notably, 20 participants (5.1%) admitted to not caring about their oral hygiene. The frequency of tooth brushing varied, with 162 (41%) brushing once a day, 170 (43%) twice a day, 45 (11%) three times a day, and 16 (4.1%) never brushing. Mouthwash usage was reported by 115 (29%), occasional use by 128 (33%), and 150 (38%) did not use mouthwash. Daily dental flossing was reported by 22%, less than 3 times a week by 23%, 10% reported flossing 3 times a week, and 44% did not use dental floss. Toothbrush replacement habits revealed that 31% replaced their toothbrush every three months, 29% every six months, 9.7% annually, and 30% whenever the opportunity arose. Participants reported varying durations for mouth cleaning, with 33% taking 1 minute, 43% taking 2 minutes, 16% taking more than 2 minutes, and 8.4% being unsure. As for bad breath perception, 77% reported not having bad breath, while 23% acknowledged experiencing it (Table 2).

**Table 2:** Participant oral hygiene practice ^1^N (%): number (percentage), and the total number of participants is 393.

Characteristic	N (%)^1^
Does taking care of oral hygiene prevent some diseases?	
No	4 (1.0%)
Yes	372 (95%)
I don't know	17 (4.3%)
When was your last visit to the dental clinic?	
Within less than three months	109 (28%)
Within 3-6 months	92 (23%)
During the past year	114 (29%)
I don't remember	78 (20%)
When did you start taking care of your oral hygiene	
In childhood	105 (27%)
In adolescence	141 (36%)
At the age of 20	82 (21%)
At the age of 30	22 (5.6%)
After the age of 30	23 (5.9%)
I don't care about my oral hygiene	20 (5.1%)
How many times do you brush your teeth per day?	
Never	16 (4.1%)
Once	162 (41%)
Twice	170 (43%)
Three times	45 (11%)
Do you use mouthwash?	
Yes	115 (29%)
Sometimes	128 (33%)
No	150 (38%)
How often do you use dental floss?	
Daily	87 (22%)
Less than three times a week	92 (23%)
Three times a week	40 (10%)
I do not use dental floss	174 (44%)
When do you change your toothbrush?	
Every three months	121 (31%)
Every six months	115 (29%)
Every year	38 (9.7%)
Whenever the opportunity arises	119 (30%)
How long does it take to clean your mouth?	
One minute	130 (33%)
Two minutes	168 (43%)
More than two minutes	62 (16%)
I don't know	33 (8.4%)
Do you have bad breath?	
No	303 (77%)
Yes	90 (23%)

Experiencing bad breath was associated with having acute tonsillitis; however, changing toothbrushes every three months and having dental visits within less than three months was associated with having acute tonsillitis (p<0.05) (Table 3).

**Table 3:** Association between acute tonsillitis and oral hygiene ^1^Response is expressed as a number (percentage). The participants who had tonsillitis (Yes) = 250. The participants who did not have tonsillitis (No) = 143. ^2^Fisher's exact test; Pearson's chi-squared test.

Question	Tonsillitis^1^		p-Value^2^
	No	Yes	
Do you think that taking care of oral hygiene prevents some diseases?			0.087
No	0 (0%)	4 (1.6%)	
Yes	140 (98%)	232 (93%)	
I don't know	3 (2.1%)	14 (5.6%)	
When was your last visit to the dental clinic?			0.014
Within less than three months	31 (22%)	78 (31%)	
Within 3-6 months	33 (23%)	59 (24%)	
During the past year	39 (27%)	75 (30%)	
I don't remember	40 (28%)	38 (15%)	
When did you start taking care of your oral hygiene			>0.9
In childhood	41 (29%)	64 (26%)	
In adolescence	51 (36%)	90 (36%)	
At the age of 20	29 (20%)	53 (21%)	
At the age of 30	8 (5.6%)	14 (5.6%)	
After the age of 30	8 (5.6%)	15 (6.0%)	
I don't care about my oral hygiene	6 (4.2%)	14 (5.6%)	
How many times do you brush your teeth per day?			>0.9
Never	6 (4.2%)	10 (4.0%)	
Once	60 (42%)	102 (41%)	
Twice	59 (41%)	111 (44%)	
Three times	18 (13%)	27 (11%)	
Do you use mouthwash?			0.4
Yes	37 (26%)	78 (31%)	
Sometimes	46 (32%)	82 (33%)	
No	60 (42%)	90 (36%)	
How often do you use dental floss?			0.4
Daily	25 (17%)	62 (25%)	
Less than three times a week	36 (25%)	56 (22%)	
Three times a week	16 (11%)	24 (9.6%)	
I do not use dental floss	66 (46%)	108 (43%)	
When do you change your toothbrush?			0.035
Every three months	34 (24%)	87 (35%)	
Every six months	39 (27%)	76 (30%)	
Every year	16 (11%)	22 (8.8%)	
Whenever the opportunity arises	54 (38%)	65 (26%)	
How long does it take to clean your mouth?			0.8
One minute	44 (31%)	86 (34%)	
Two minutes	61 (43%)	107 (43%)	
More than 2 minutes	24 (17%)	38 (15%)	
I don't know	14 (9.8%)	19 (7.6%)	
Do you have bad breath?			0.015
No	120 (84%)	183 (73%)	
Yes	23 (16%)	67 (27%)	

Among the 250 participants with acute tonsillitis, 13 participants (5.2%) reported never having acute tonsillitis when they were over 20 years, while 49 (19.6%) experienced it twice, 11 (4.4%) three times, and 96 (38.4%) more than three times. When seeking a medical diagnosis for acute tonsillitis, 99 participants (40%) visit a doctor each time. Of those diagnosed, 198 (79%) received antibiotic treatment, with 42 (21%) receiving it once, 58 (29%) twice, and 39 (19%) three times. Thirty-eight (15%) had a throat swab taken among those diagnosed with acute tonsillitis. A significant majority, 87%, experienced fever during acute tonsillitis, and 238 (95%) had difficulty swallowing. Regarding the impact on daily life, 41 (16%) reported not missing any work/school days, 84 (34%) missed three days or less, and 41 (16%) missed more than three days (Table 4). 

**Table 4:** Characteristics of participants with acute tonsillitis ^1^N (%): number (percentage), and the total number of participants is 393.

Characteristic	N (%)^1^
How many times have you had acute tonsillitis when you were over 20 years old?	
Not once	13 (5.2%)
Twice	49 (19.6%)
Thrice	11 (4.4%)
From four to five times	50 (20%)
More than five times	46 (18.4%)
I don't remember	81 (32.4%)
Did you visit a doctor for a diagnosis each time?	
No	151 (60%)
Yes	99 (40%)
Were you treated with antibiotics when diagnosed?	
No	52 (21%)
Yes	198 (79%)
How many times has an antibiotic been prescribed to treat acute tonsillitis?	
Once	42 (21%)
Twice	58 (29%)
Thrice	39 (19%)
From three to five times	24 (12%)
More than five times	40 (20%)
Unknown	47
Was a throat swab taken during the diagnosis?	
No	212 (85%)
Yes	38 (15%)
Did you experience a fever when you were sick?	
No	32 (13%)
Yes	218 (87%)
Did you have any problems swallowing when you were sick?	
No	12 (4.8%)
Yes	238 (95%)
On average, how many days did you miss work/school due to acute tonsillitis each time?	
Not a day	41 (16%)
Three days or less	84 (34%)
From three to five days	31 (12%)
From seven to 14 days	10 (4.0%)
I don't remember	84 (34%)

**Figure 1:**
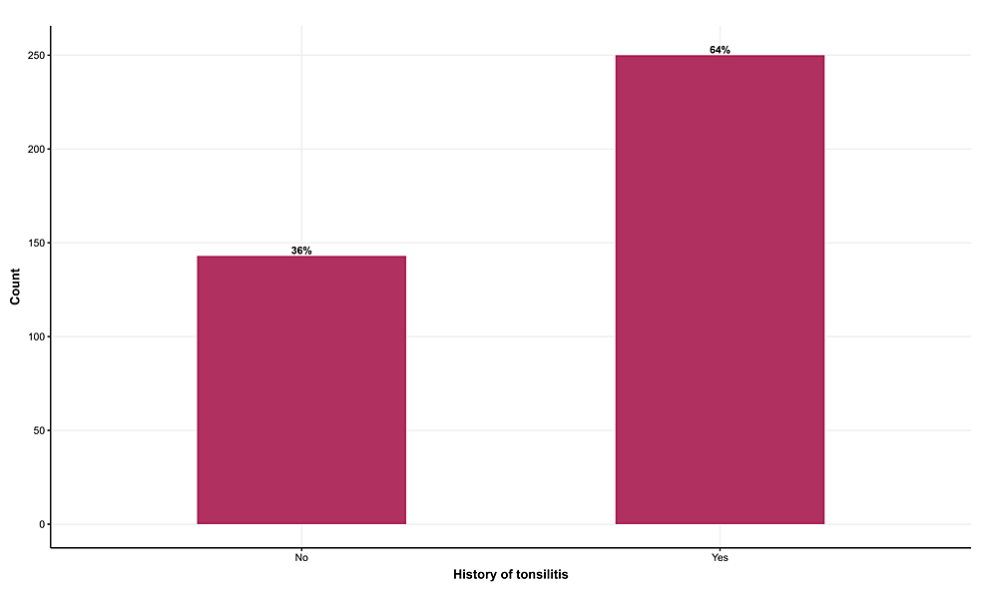
Prevalence of acute tonsillitis

Approximately 64% of the participants had or experienced acute tonsillitis (Figure 1). 

**Figure 2:**
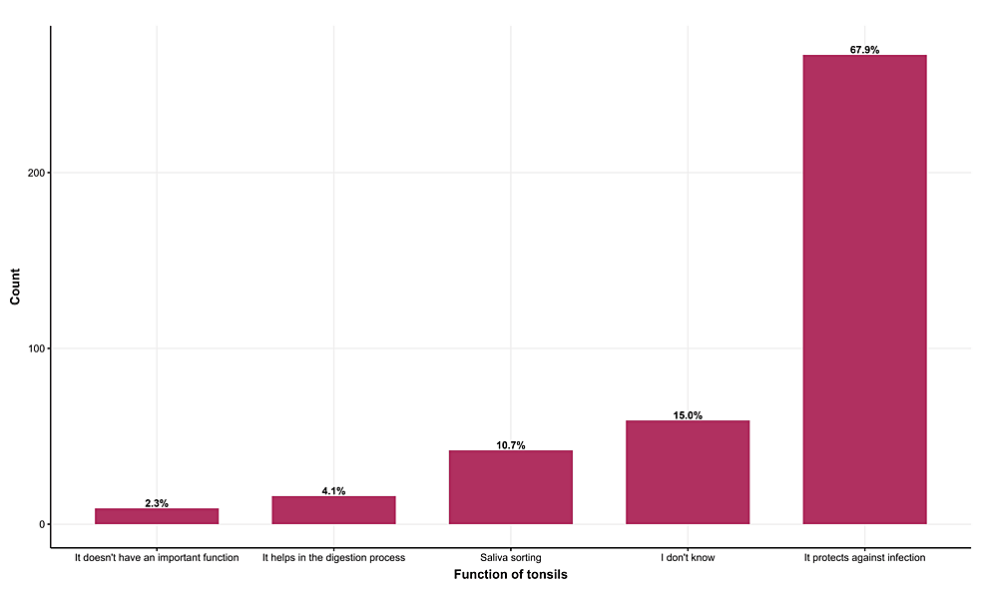
Participants' perspectives on the functionality of tonsils

Most participants (67.9%) recognize the immunologic function of tonsils. However, 15% needed to recognize its role (Figure 2).

Experiencing bad breath (OR: 2.11, 95% CI: 1.23, 3.70) was linked to a higher risk of acute tonsillitis while adopting less frequent toothbrush change was linked to a lower risk (OR: 0.54, 95% CI: 0.30, 0.94) (Table 5).

**Table 5:** Oral hygiene determinants of acute tonsillitis OR = odds ratio, CI = confidence interval. ^1^Fisher's exact test; Pearson's chi-squared test.

Characteristic	OR	95% CI	p-Value^1^
Bad breath			
No	-	-	
Yes	2.11	1.23, 3.70	0.008
Frequency of toothbrush change			
Every three months	-	-	
Every six months	0.81	0.45, 1.44	0.5
Every year	0.47	0.21, 1.04	0.059
Whenever the opportunity arises	0.54	0.30, 0.94	0.029
Last visit to the dental clinic			
During the past year	-	-	
Within less than three months	1.39	0.77, 2.52	0.3
Within 3-6 months	0.93	0.52, 1.69	0.8
I don't remember	0.56	0.30, 1.04	0.067

## Discussion

This cross-sectional study aimed to assess the prevalence of acute tonsillitis and the relationship between acute tonsillitis and oral hygiene among the population in Taif City. Males represent about 53% of our study participants, and the majority were in the age group 20-30 years (54%). Ninety percent were Saudi and 70% had a university level of education. 

The frequency of acute tonsillitis reported by our participants is about 64%; about 38% reported experiencing acute tonsillitis more than three times. A study by Hidaya et al. among the Saudi pediatric population found that the incidence of acute tonsillitis was higher among the age group 6-12 years (69%), followed by 13-18 years (18%), and finally the age group 4-5 years (17%). The occurrence of tonsillitis was found to vary according to socioeconomic status, where the incidence decreases with increasing economic status. The incidence of tonsillitis in the low-income group was 66%, the middle-income group was 34%, and 7% among the high-income group [16]. A retrospective study that investigated the incidence of tonsillitis in the Al-Baha region, Saudi Arabia, reported that the incidence is more observed among males (60%) than females (40%). The most commonly isolated organisms were GAS infections, group A beta-hemolytic Streptococci (*Streptococcus pyogenes*) [17]. 

A prospective study in Saudi Arabia surveying children with acute tonsillitis with throat swabs found evidence of GAS infection in about 40% of participants, and less than 50% were sensitive to penicillin [18]. Nevertheless, a review article reported the pooled prevalence of 37% of GAS infection among children. It was observed that GAS infection and carriage among under-five children was less than that among older children, with a pooled prevalence of 24% among those under five [19]. 

In a retrospective study in Libya, the prevalence of tonsillitis among patients who came with a sore throat to otolaryngology clinics was found to be 34%, and the majority of tonsillitis patients were females (65%), from the age group 15-45 years (48%) [20]. 

Regarding oral hygiene practices by our participants, 95% believed that taking care of oral hygiene can prevent some diseases. The majority (43%) reported brushing their teeth twice per day. The majority (38%) didn’t use mouthwash, and 44% didn’t use dental floss. Most of our participants (43%) take about 2 minutes to brush their teeth. A review article concluded that there is a relationship between oral hygiene and CT [21]. A case-control study conducted in Iraq found a strong positive correlation (p < 0.001) between dental caries and CT in children [22]. 

The majority of our study participants (79%) indicated that they were treated with antibiotics when diagnosed with acute tonsillitis, and 20% had antibiotics prescribed more than five times. Nevertheless, more than three-quarters (85%) got the diagnosis of acute tonsillitis on a clinical basis with no throat swab taken for the diagnosis. Regarding symptomatology of acute tonsillitis, 87% of our participants experienced febrile episodes, and 95% had difficulty swallowing when they were sick. However, 34% missed about three days of school/work as a consequence of their illness. In the cross-sectional study by Hidaya et al. in Al-Riyadh, fever was reported in 74% of participants. Another symptom observed was odynophagia, which was reported in 33% of the tonsillitis patients [17]. The aim of the treatment in tonsillitis cases is to decrease symptoms (pain and fever) and to decrease complications. Thus, the treatment is usually supportive, especially with viral infections. GAS infection could be treated with penicillin; if there is resistance or allergy to penicillin, cephalosporins and macrolides are good alternatives. Corticosteroids are helpful in case of infectious mononucleosis pharyngitis [23,24]. However, an interesting meta-analysis article by Mirza et al. found a statistically significant deficiency of vitamin D in patients with recurrent tonsillitis [25]. 

This study is limited by the pure cross-sectional approach with no involvement of a comparative control group. Yet, the relatively larger sample size could adjust for any potential bias that might be encountered in this study.

## Conclusions

In our survey, 64% reported acute tonsillitis prevalence, with most acknowledging the impact of oral hygiene. Despite quick teeth brushing being common, these habits may contribute to the prevalence. Participants who had bad breath were at more risk for developing acute tonsillitis. Reducing the frequency of toothbrush changes was associated with a lower risk of having acute tonsillitis.

Antibiotic treatment was common, especially with 20% prescribed more than five times. Surprisingly, over three-quarters received a clinical diagnosis without a throat swab. Symptomatically, 87% experienced febrile episodes, and 95% had difficulty swallowing. Acute tonsillitis, seemingly simple, led to frequent school and work absences, emphasizing the need for heightened attention and further research in this area. Addressing oral hygiene practices could be a key focus for preventative measures.

## Appendices

**Table 6:** Questionnaire

	Do you agree to participate in this survey?	Yes ​​☐​	No ​​☐​			
	Age	From 20 to 30 years old ​☐​	From 31 to 40 years old ​☐​	From 41 to 45 years old ​☐​	Other ☐	
	Gender	Male ​​☐​	Female ​☐​			
	Nationality	Saudi ​​☐​	Non-Saudi ​​☐​			
	Marital status	Single ​​☐​	Married ​​☐​	Divorced ​​☐​	Widowed ​​☐​	
	Educational level	High school or lower ☐	University ☐	Postgraduate studies ☐		
What is the function of the tonsils?	It protects against infection. ​☐​	It helps in the digestion process. ​​☐​	Saliva sorting. ​​☐​	It doesn't have an important function. ​​☐​	I don't know. ​​☐​	
	Do you think tonsillitis is common?	Yes. ​​☐​	No. ​​☐​	I don't know. ​​☐​		
	Do you think tonsillitis leads to serious complications?	Yes. ​​☐​	No. ​​☐​	I don’t know ​​☐​		
	Do you think that taking care of oral hygiene prevents some diseases?	Yes. ​​☐​	No. ​​☐​	I don't know. ​​☐​		
	When was your last visit to the dental clinic?	Within less than 3 months. ​​☐​	Within 3-6 months. ​​☐​	During the past year. ​​☐​	I don't remember. ​​☐​	
When did you start taking care of your oral hygiene?	In childhood. ​​☐​	In adolescence. ​​☐​	At the age of twenty. ​​☐​	At the age of thirty. ​​☐​	After the age of thirty. ​​☐​	I don't care about my oral hygiene. ​​☐​
	How many times do you brush your teeth per day?	Once. ​​☐​	Twice. ​​☐​	Three times. ​​☐​	Never. ​​☐​	
	Do you use mouthwash?	Yes ​​☐​	No ​​☐​	Sometimes ​​☐​		
	How often do you use dental floss?	Daily ​​☐​	3 times a week ​​☐​	Less than 3 times a week ​​☐​	I do not use dental floss ​​☐​	
	When do you change your toothbrush?	Every 3 months ​​☐​	Every 6 months ​​☐​	Every year ​​☐​	Whenever the opportunity arises ​​☐​	
	How long does it take to clean your mouth?	One minute ​​☐​	Two minutes ​​☐​	More than two minutes ​​☐​	I don't know ​​☐​	
	Do you have bad breath?	Yes ​​☐​	No ​​☐​			
	Have you ever had tonsillitis?	Yes ​​☐​	No ​​☐​			
​​​	How many times have you had tonsillitis when you are over twenty years old?	Twice or less. ​​☐​	From 3 to 5 times. ​​☐​	5 times or more. ​​☐​	I don't remember. ​​☐​	Not once. ​​☐​
	Did you visit a doctor for diagnosis each time?	Yes. ​​☐​	No. ​​☐​			
	Were you treated with antibiotics when diagnosed?	Yes ​​☐​	No ​​☐​			
	If yes, how many times has an antibiotic been prescribed to treat tonsillitis?	Once ​​☐​	Twice ​​☐​	3 times ​​☐​	From 3 to 5 times ​​☐​	More than 5 times ​​☐​
	Was a throat swab taken during the diagnosis?	Yes ​​☐​	No ​​☐​			
	Did you experience a fever when you were sick?	Yes. ​​☐​	No. ​​☐​			
	Did you have any problems swallowing when you were sick?	Yes. ​​☐​	No. ​​☐​			
	On average, how many days did you miss work/school due to tonsillitis each time?	3 days or less. ​​☐​	From 3 to 5 days. ​​☐​	From 7 to 14 days. ​​☐​	I don't remember. ​​☐​	Not a day. ​​☐​
